# Intra-Population Genetic Variation in the Temporal Pattern of Egg Maturation in a Parasitoid Wasp

**DOI:** 10.1371/journal.pone.0045915

**Published:** 2012-09-27

**Authors:** Eric Wajnberg, Christine Curty, Mark Jervis

**Affiliations:** 1 INRA, Sophia Antipolis, France; 2 Cardiff School of Biosciences, Cardiff University, Cardiff, United Kingdom; University of California, Berkeley, United States of America

## Abstract

Parasitoid wasps are taxonomically and biologically extremely diverse. A conceptual framework has recently been developed for understanding life-history evolution and diversification in these animals, and it has confirmed that each of two linked life-history traits – the mode of larval development and the temporal pattern of egg maturation – acts as an organiser of life-history. The framework has been predicated on the assumption that there exists sufficient genetic variation in the latter trait to allow it to be shaped by natural selection. Focusing on the parasitoid wasp *Trichogramma brassicae*, our aim was to test the validity of that assumption, using established quantitative genetic methods. We demonstrate the existence of a statistically significant degree of intra-population polygenic variation in the temporal pattern of egg production within the wasp population we studied. Furthermore, our results, together with published data on clinal variation in the egg maturation pattern of another species, suggest that intra-specific evolutionary shifts in the temporal pattern of egg maturation of parasitoid wasps can result from a change in allocation to egg production either before, or very shortly after adult emergence, without there being an accompanying change in lifetime fecundity. As well as opening new avenues of research into the reproductive strategies, behaviour, community organisation and biological control potential of parasitoid wasps, this discovery also has implications for studies of life-history evolution and diversification in insects generally.

## Introduction

Parasitoid wasps are among the most intensively studied of all insects. This is attributable to their extremely high taxonomic diversity, their near-ubiquity among terrestrial insect communities, their top-down role in host population dynamics (which makes them economically as well as ecologically important), and the ease with which they can be reared, cultured and experimented with [1]. Despite most species sharing the same basic life-cycle, they show a striking degree of inter-specific diversity in their life-histories [1]–[2]. Comparative studies have sought to make sense of that diversity by testing for statistical relationships among life-history traits. The recorded correlations have provided insights into the selection pressures responsible for trait evolution [1], [3]–[6]. Hypotheses and inferences regarding those correlations have so far been predicated on the assumption that much of the phenotypic variation in the traits is under genetic control and can therefore be moulded by natural selection. Genetic variation has been documented for several parasitoid life-history traits, and heritability measures have been obtained for development time, body size, fecundity and adult longevity, resistance against the host’s encapsulatory defence mechanism, juvenile survival, progeny sex ratio and mating system (polyandry) [7]–[9]. The first four of these traits are included in the two widely recognised conceptual frameworks of parasitoid life-history evolution and diversification, one of which is centred on the mode of larval development, the other is based on the overall temporal pattern of egg maturation [1], [10].

Those frameworks have recently been combined into a single holistic perspective, due to the discovery that the two ‘organising’ traits are themselves linked and have several correlates in common, namely: relative egg yolk-richness, female life-span, pre-adult life-span and maximum egg load; for explanations of the functional significance of the relationships, see [1]. Currently, except for circumstantial evidence regarding the second (see below), nothing is known about genetic variation of the organising traits within natural populations and thus the potential for those traits to evolve in response to natural selection. This is a major lacuna in knowledge, given not only the traits’ conceptual significance (particularly to life-history theory and behavioural ecology) but also their practical importance (to insect pest management).

Phenotypically, the mode of larval development (idiobiosis *vs* koinobiosis) is a discontinuous, phylogenetically highly constrained and intra-specifically unvarying trait, whereas the overall lifetime pattern of oogenesis is a continuous, phylogenetically much less constrained and intra-specifically variable trait [1], [11]. Thus, the latter is the obvious of the two to focus on from the standpoint of genetic variability and the trait’s capacity to evolve in response to natural selection. Furthermore, the idiobiosis *vs* koinobiosis dichotomy in mode of larval development is unique to insect parasitoids, whereas the egg maturation strategy is known also to be an organising trait in the life-history evolution and diversification of non-parasitoid insects, notably Lepidoptera [12]–[14]. Thus, insights gained from an investigation of genetic variability in the temporal pattern of egg maturation will have broader entomological relevance.

In parasitoid wasps, intra-specific phenotypic variation in the temporal pattern of egg maturation is partly linked to body size [6], [15] and partly due to as yet unconfirmed causes, although theoretical modelling has identified host availability as one important driver [16]–[17]. Currently, the only evidence that such phenotypic variation has a genetic component consists of a difference, sustained over several generations, in initial egg load (the number of mature eggs present when the adult emerges), but not lifetime realized fecundity, between laboratory cultures of the parasitoid wasp *Asobara tabida* Nees. The cultures had been derived from geographically widely separated field populations along a north-south cline [18]. The fact that the initial egg load differences were consistently maintained in the laboratory cultures strongly suggests that the populations have, in the field, diverged genetically with respect to the overall temporal pattern of egg maturation.

Using a simple measure of the degree of concentration of egg production into early adult life, and employing two well tried-and-tested quantitative genetic methods to a sample population of a commonly studied and economically important parasitoid wasp species, we established that significant genetic variation does occur in the investigated trait. Furthermore, we obtained evidence that intra-specific evolutionary shifts in the temporal pattern of egg maturation of parasitoid wasps involve a change in allocation to egg production, taking place around the time of adult eclosion. Our results, in the light of current knowledge concerning parasitoid egg maturation strategy generally, have important implications for research into the reproductive strategies, behaviour, community organisation, and biological control potential of parasitoid wasps. They also have major relevance to studies of life-history evolution and diversification in other insects (Lepidoptera) in which the temporal pattern of egg maturation is an organising trait [12]–[13].

## Materials and Methods

### Insects

The species used in this study was a chalcidoid wasp, *Trichogramma brassicae* Bezdenko ( = *T. maidis* Pintureau & Voegelé) (Hymenoptera: Trichogrammatidae), a polyphagous egg parasitoid whose preferred hosts are the eggs of the European Corn Borer, *Ostrinia nubilalis* (Hübner) (Lepidoptera: Pyralidae) [19], [20]. The strain used in the experiment originated from ca. 30 mated females reared from parasitized *O*. *nubilalis* egg masses taken from a corn field in Southern France in June 2010. The strain was maintained as separate lines under laboratory conditions for ca. 6 months in eggs of the Mediterranean Flour Moth, *Ephestia kuehniella* Zeller, at 25°C and a 12 h:12 h light:dark regime. No specific permits were required for the described study.

### Reproductive Concentration Index (RCI)

To date, two ‘shorthand’ measures of variation in the temporal pattern of egg maturation have been employed in empirical studies of parasitoid reproductive strategy [21]: (a) the initial egg load – the number of mature eggs carried by a female at the very start of adult life (e.g. [15], [18]), and (b) the Ovigeny Index (OI) – the initial egg load divided by the lifetime potential fecundity [1], [6], [11]–[12], [22]–[23]. Here, we use an additional measure, the Reproductive Concentration Index (RCI) which, like the OI, quantifies the relative degree to which females concentrate egg production into very early adult life. While the OI is based on the initial egg load of the females upon adult emergence, the RCI is based on the egg load of unfed adult females shortly after their emergence. Just like the OI, the higher the RCI index value, the greater the fraction of the lifetime complement of eggs that is matured during the very early phase of adult life. The essential difference between these two indices is that the numerator of the OI measures the investment in eggs made, from teneral (*i.e.*, carried-over) resources, by the time of adult emergence, whereas the numerator of the RCI additionally measures the investment made during a brief phase after emergence.

The age-specific oogenesis and fecundity schedules of parasitoid wasps and other oviparous insects tend to be positively skewed: following the onset of the reproductive phase of life there is a rise in egg production and oviposition, followed by a slow decline due to reproductive senescence (the obvious exceptions to this overall pattern being species that emerge with the whole of their lifetime egg complement in a fully mature state). *T. brassicae*’*s* egg production schedule conforms to the general pattern. Specifically, peak egg production occurs at about 24 h of age, having risen sharply (see below), and from the peak onwards it declines rapidly ([24]; reported as *T*. *maidis*). Graphically, such a pattern is termed the ‘triangular fecundity function’ [25]–[26]. Intra-specific phenotypic alterations in parasitoid wasp age-specific fecundity schedules – for example those brought about by a change in host density regime – typically involve changes in curve skewness (*e.g.*, see Fig. 3 in [18]), and it is reasonable to assume that alterations in the age-specific oogenesis schedule also can occur. As a tool for establishing the existence of genetic variation in the age-specific oogenesis schedule when applying quantitative genetics techniques, the RCI was, from our point of view, preferable to either the OI or the initial egg load for two practical reasons: (a) in insects, variation in the curve (*i.e.*, change in skewness) might not necessarily be accompanied by variation in initial egg load (the numerator of the OI), as is evident at both the intra- and the inter-specific level [11], [23], [27], and (b) obtaining measurements of early-life egg production is logistically simpler in the case of the RCI, as parasitized eggs do not need to be continuously monitored for emerging wasps. If the OI were used, such monitoring would be impracticable due to the very large numbers of adult wasps necessary for quantitative genetic study.

Due to the destructive effects of dissection, measuring early-life egg load in individual parasitoids precludes estimating lifetime potential fecundity in very small-bodied species such as *T. brassicae* (in such wasps, the smallest of the developing oocytes in the ovarioles cannot be counted precisely). Therefore, to compute the RCI we examined every batch of parasitized eggs every morning (*i.e.*, every 24 h), for emerged female progeny. These wasps, all of which were deprived of food, were collected and immediately killed by placing them in a freezer at −20°C. The wasps were subsequently thawed and dissected to count the number of mature eggs contained within their reproductive systems. The number of counted mature eggs was divided by the overall lifetime realized fecundity, measured using another group of females. Females from that second set had been mated, reared individually under standardized conditions (25°C, 12 hL:12 hD) and given both abundant food (diluted commercial honey) and unlimited opportunities to lay eggs over their entire lifespan by providing them with an excess of eggs of *E. kuehniella* (additional host eggs were provided every second day, until the wasp died). A daily excess of hosts was intended to exhaust the ovaries’ supply of oocytes [28]. Lifetime realized fecundity was then measured by counting the cumulative number of host eggs that had turned black after five days following parasitism, indicating that the hosts had definitely been parasitized. Under a very low female/host ratio, as was the case in our experiment, superparasitism of hosts is a very rare occurrence in *T. brassicae* [29] and only a very small fraction of attacked eggs abort before turning black (less than one percent; unpublished observations). Hence, the number of hosts parasitized was a reasonable estimate of the number of eggs actually laid by the females, *i.e.*, their lifetime realized fecundity. Under conditions of abundant food and unlimited hosts, the latter is routinely used to approximate lifetime potential fecundity [1], [6]. To ascertain that all females had actually mated, the presence of daughters in the progeny was always verified.

We should stress that the first 24 h of adult life represents a very small portion of the *T. brassicae* females’ total lifetime duration. By the time females were sampled for dissection, they had attained at most only 6.5% of their potential lifespan (the mean value for the latter, measured in the second group of females, was 15.296±0.0422 [SE] days). Because some of the females dissected at the end of the 24 h monitoring period may have been actually less than 24 h old (possibly even less than 1 h old in a few cases) when killed, the RCI is likely to measure largely variation in the pre-peak phase of the oogenesis curve, rather than in the 24 h peak itself.

The RCI’s utility – relative to the OI – for making inter-specific comparisons among species has yet to be ascertained. However, provided that egg load is measured during the very early phase of adult life (as was done here), the two indices are likely to be positively correlated inter-specifically. Even if such a correlation turns out to be very weak or non-existent, it would not detract from the utility of the RCI in revealing shifts in the temporal pattern of egg maturation.

### Quantification of Genetic Variation

Different experimental protocols can be used to quantify genetic variation in quantitative phenotypic traits [8]. They are most commonly based on either of two types of analysis: (1) Isofemale lines analysis (‘family analysis’; [30]–[32]) in which trait variation between progeny derived from individual, randomly inseminated females is compared, and (2) Parent-offspring regression (‘mother-daughter regression’) in which the genetic transmissibility of the trait is assessed over two successive generations. These complementary approaches have previously been successfully applied to *T*. *brassicae* and other parasitoid wasps having a haplodiploid mode of sex determination (*e.g.*, [29], [33]–[36]; see [8], for a review), and we use them here to quantify genetic variability in the RCI. A mother-daughter regression analysis was performed over two successive generations, using the average of several offspring collected from each mother. At the following generation, the daughters represented different isofemale lines, which were then compared using a family analysis [8].

**Figure 1:**
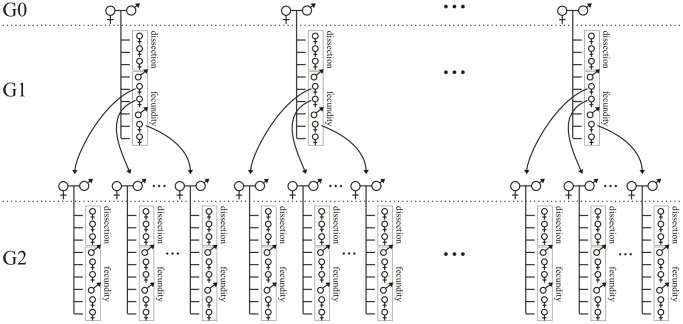
Representation of the design used to quantify genetic variation in the RCI in *Trichogramma brassicae*. The Reproductive Concentration Index (RCI) quantifies the relative degree to which females concentrate egg production into very early life. Females dissected up to 24 h after emerging from the host egg were used to compute the numerator of the RCI (*i.e.*, egg load). By the time these females were sampled, they had attained at most only 6.5% of their potential lifespan. The remaining females provided data to estimate the denominator (*i.e.*, lifetime realized fecundity, measured under conditions of available food and an excess of hosts). With this protocol, the genetic variation in RCI was estimated in two ways: (1) by computing/testing the regression between G1 and G2 (over two successive generations; *i.e.*, a ‘mother-daughter regression analysis’), and (2) by comparing the different families (only three of them are represented in this figure) at the G2 generation (a so-called ‘family analysis’).

Fifteen mated females, taken at random from the mass-reared population, were used to establish 15 isofemale lines (*i.e.*, families) at the grandmother (*i.e.*, G0) generation. At the following mother generation (G1), several females were dissected upon emergence, and others were kept individually with a male for mating and their lifetime fecundity estimated. Also, one hind tibia of each dissected female was mounted on a slide and its size measured with an eyepiece micrometer mounted on a standard stereomicroscope to an accuracy of 0.01 µm. The measurement served as a proxy for adult size [37]. Finally, the longevity of the females used in estimating fecundity was also recorded to an accuracy of one day. Those females were also used as mothers to found a G2 (daughter) generation, thus producing several subfamilies per family. The process was then repeated at G2, providing us with RCI values for the next generation in each family. Because, for one particular family, measurement of RCI was possible only at G2, there were 14 families at G1 and 15 at G2. The entire experimental procedure is summarized in Figure 1.

Lifetime realized fecundity was measured only for females that had been mated with one of their brothers, in order to increase the genetic relatedness between females at the mother and the daughter generations. On average (± SE), at G1, lifetime realized fecundity and longevity were measured for 3.14±0.23 (range: 2–5) daughters per mother (giving a total of 44 females), while 5.64±0.25 (range: 3–6) daughters per mother were dissected and their tibia length measured (giving a total of 79 females). At G2, lifetime realized fecundity and longevity were measured for 3.60±0.13 (range: 1–5) daughters per mother (giving a total of 173 females), while 5.31±0.15 (range: 2–6) daughters per mother were dissected and their tibia length measured (giving a total of 255 females). All replicates were randomly distributed over the full experimental period at both generations.

### Statistical Analysis

‘Twenty-four hour’ egg load and lifetime realized fecundity are each likely to be correlated with female body size [6], [15]–[16]. Consequently, recorded genetic variation in RCI might be solely the result of genetic variation in female body size. Furthermore, longer-lived females will very likely have a higher overall lifetime realized fecundity, since most of them will lay eggs until death, and so recorded genetic variation in RCI could be solely the result of genetic variation in female longevity. In order to circumvent these problems, we corrected our ‘24 h’ egg load counts by removing the effect of female size in a ’24 h’ egg load *vs* body size linear regression, and corrected our fecundity estimates by removing the effect of female longevity in a fecundity *vs* longevity linear regression. The corrections involved subtracting, from the measured egg load, the egg load predicted by the egg load *vs* body size linear regression. Similarly, we subtracted, from the measured lifetime realized fecundity, the value predicted from the fecundity *vs* longevity linear regression. All measured females were used to compute the corresponding regressions. After such correction, there no longer existed, by definition, a relationship either between egg load and female body size or between fecundity and female longevity. We performed both corrections separately for G1 and G2 since the variables were not measured concurrently for these two generations. Hence, the RCIs computed, post-correction, were for females having an average size and an average longevity. Therefore, any observed significant genetic variation in RCI that was evident post-correction could not have been the result of significant genetic variation in either body size or their longevity. There is no reason to suspect that RCI estimated in this way would not follow a normal distribution (applying Shapiro’s and Bartlett’s tests did not reveal significant deviation from normality or homoscedasticity among families; all P-values exceeded 0.18). Therefore, both the statistical test of the mother-daughter relationship over two successive generations, and the calculation of the difference between the different families at the daughter generation, using each subfamily as a replicate, were performed using standard parametric methods, *i.e.*, a simple linear regression and a one-way ANOVA, respectively.

### Ovigeny Index (OI)

We investigated this life-history variable because values have not previously been obtained for *T. brassicae*. Measuring OI is becoming routine in studies of parasitoid wasp reproduction, irrespective of the focus of interest. We measured initial egg load in an additional group of 38 females less than 1 h after they had emerged. Using the mean of the data gathered, and dividing it by the mean for our estimate of lifetime fecundity for the aforementioned second group of females (pooled over the mother and the daughter generations), we were able to estimate the OI.

## Results

The experimental data were first analysed by pooling the results obtained for both mothers and daughters. The results show that, on average, *T*. *brassicae* females had an RCI of 0.3099±0.0090 (± SE).

**Figure 2:**
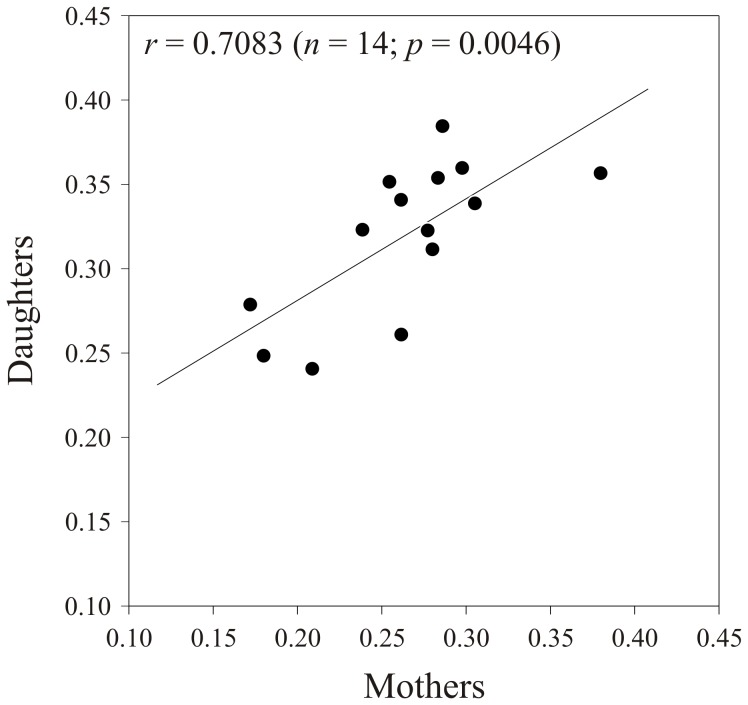
Mother-daughter regression analysis of the Reproductive Concentration Index (RCI) for *Trichogramma brassicae* females. Values of the daughters are the averages of several offspring collected from each mother. The regression line and *p*-value are shown. The equation of the regression line is y = 0.6025x+0.1607. R^2^ = 0.502.

**Figure 3:**
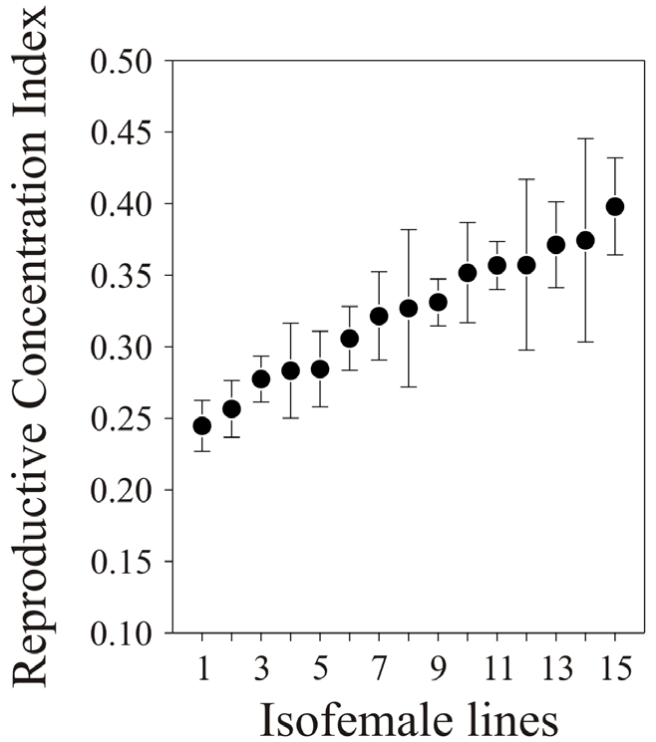
Average (±SE) Reproductive Concentration Index (RCI) quantified for 15 isofemale lines of *T*. *brassicae* females. Lines are ordered according to increasing average values of the trait studied. Sample sizes for the 15 lines, from left to right, are: 4, 4, 3, 2, 4, 2, 2, 2, 3, 3, 5, 3, 5, 3 and 3. The inter-line variation is statistically significant (ANOVA: *F*_14,33_ = 2.08, *P* = 0.0417).

Figure 2 presents the mother-daughter regression analysis of our RCI data. There is a highly significant relationship between the RCI values obtained for the mothers and those obtained for their daughters (*P* = 0.0046). The *R* ^2^ value indicates that over 50% of the variability in daughter values is explained by mother values (see Figure 2). Furthermore, the ANOVA performed on data for the daughter generation revealed significant variation in RCI between the different families that were compared (*F*_14,33_ = 2.08, *P* = 0.0417); their average values are plotted in Figure 3.

Both results strongly show that, in the *T*. *brassicae* population we examined, there is a significant genetic component to phenotypic variability observed in RCI. Some genotypes are leading females to mature a higher proportion of their lifetime complement of eggs prior to emergence than others.

Interestingly, genetic variation in RCI seems to be mainly attributable to the genetic variation that exists in the egg load females carry at around the start of adult life (mother-daughter regression: r = 0.577, *P* = 0.03; family analysis: F_14,33_ = 2.99, *P* = 0.0048), and not to genetic variation in lifetime fecundity (mother-daughter regression: r = 0.517, *P* = 0.581; family analysis: F_14,33_ = 1.82, *P* = 0.078).

We obtained an OI value of 0.196 for *T. brassicae*. Hence, females emerge with some mature eggs, and these constitute less than a fifth of the eggs that could potentially be laid by the females during their lifetimes. This species can thus be described as ‘strongly’ synovigenic [1], [6], [21] because females emerge, on average, with less than half of their lifetime potential complement of eggs already mature.

## Discussion

Our results demonstrate the existence of a statistically significant amount of genetically determined variation in the temporal pattern of egg maturation in a parasitoid wasp. Shifts in the insects’ oogenesis schedule were measured using the Reproductive Concentration Index (RCI) which, for purely practical reasons in this study, was regarded as preferable to the Ovigeny Index (OI). A consideration of OI is, nevertheless, highly pertinent to our discussion, insofar as close parallels can be drawn between the OI and the RCI as regards their utility in understanding insect life-history variation. Like the OI, the RCI measures the relative degree to which egg production is concentrated into very early adult life. Similarly, it can be used as an intra-specific measure of the relative degree of carry-over, from the larva, of physiological resources utilized by the adult in egg production and somatic maintenance. Investment of carried-over (*i.e.*, teneral) resources in early-life reproduction can be expected to occur at the expense of investment in other adult life-history traits, in accordance with general life-history theory [38]–[40]. The *T. brassicae* females used for early-life egg load measurement were deprived of food prior to dissection. Therefore, in those individuals there cannot have been an input of exogenous nutrients towards oogenesis (as would also have applied to females sampled at the precise time of emergence to calculate OI values). The egg load will have been produced entirely from teneral resources, likely at a cost to other allocations [11]–[13]. Thus, by analogy with the OI conceptual framework, our results for the RCI can be taken to have major implications for studies of the evolution and the diversification of egg maturation strategy and other life-history traits in parasitoid wasps.

In parasitoid wasps, synovigeny (*i.e.*, OI <1.0) is by far the most common egg maturation strategy [6], as is the case for *T. brassicae* and other trichogrammatids. Modelling which has examined the intra-specific relationship of OI to variation in body size has revealed different degrees of synovigeny (values of OI above zero but <<1) to be adaptations to habitat richness, specifically combinations of mean host abundance with host dispersion pattern (aggregation) [16]. Inter-specifically in parasitoid wasps, OI is positively correlated with maximum egg load (defined as the maximum number of eggs that can be contained within the females’ reproductive apparatus at any time during adult life) [1]. This points to the existence of a functional link between oviposition rate and the temporal pattern of egg maturation [1], and it implies a positive correlation between the latter trait and host availability. Parasitoid female mortality risk, insofar as it is determined by habitat-related factors (*e.g.*, predation, [41]) is another factor that could drive both inter- and intra-specific variation in egg maturation strategy [22].

Our results show that the potential exists for insect populations to respond, in terms of the age-specific oogenesis schedule and correlated traits, to directional selection brought about by changes in the aforementioned habitat-related factors. It is reasonable also to infer that the wide diversity in egg maturation strategy, seen inter-specifically among parasitoid wasps and other insects such as Lepidoptera, is at least partly the result of such selection (for underlying rationale, see [6], [11]–[14]).

It is possible that, in some evolutionary lineages, there exist constraints upon intra-specific variation in the initial egg load. These constraints are, in our opinion, more likely to operate in clades whose members typically have an initial egg load of zero, particularly where host feeding is a prerequisite for initiating oogenesis. However, the possibility that they also operate in clades where initial egg load is higher than zero (*i.e.*, that comprising the Trichogrammatidae) cannot be ruled out – our use of the RCI rather than the OI was therefore a prudent choice. *A priori*, an invariant initial egg load would not necessarily preclude either shifts in the oogenesis schedule or the trading of early-life reproduction against other traits predicted under the current conceptual framework (see [11]–[14]). Thus, generally among synovigenic parasitoid wasps, as per that framework, evolutionary scenarios can be envisaged which result in shifts in skewness of the oogenesis schedule of a population or species [14]: one involves a leftwards shift driven by improved habitat richness, *e.g.*, an increase in overall host abundance and/or a widening of the insects’ host range; another involves a rightwards shift (resulting in a flattening and widening of the oogenesis curve) driven by host scarcity (in this scenario the adverse fitness effects of time-limitation select for an increase in potential adult lifespan). Both of these scenarios are likely to be reflected in the pattern of allocation of different teneral resources [11]–[13], [42].

Many parasitoid wasps are plastic as regards the shape of their age-specific realised fecundity schedule in response to the ambient level of host availability: at high host densities egg deposition becomes concentrated into early life [23]. Whilst it is tempting to conclude that such shifts are largely, if not entirely, mediated by an alteration in the rate of oogenesis, there is currently a paucity of supportive evidence (see [43] for one postulated mechanism). Interestingly, *A. tabida*’s northern and southern clinal populations, which occur in host-poor and host-rich habitats respectively, each combine adaptive plasticity in age-specific fecundity schedule with apparently genetically fixed allocations of teneral resources to initial egg load *vs* fat, the latter being the fuel for future egg production and somatic maintenance [18]. Presumably it is because of the trade-off in allocation of resources to those two traits that the clinal populations have different estimated OIs [6], [12]. Note that the difference in the OI of *A. tabida* is entirely due to inter-population differences in initial egg load. Similarly, the genetically-based variation seen in the RCI of *T*. *brassicae* is wholly attributable to variation in very early-life egg load, as we recorded no significant genetically-based variation in the denominator of that index. Thus, based on current evidence, we can infer that intra-specific evolutionary shifts in the temporal pattern of egg maturation of parasitoid wasps can result solely from changes that occur in allocation to egg production prior to, or very shortly after, adult emergence.

Our results also have implications for insect community ecology [44], [45]. Modelling has shown that, theoretically, life-history trade-offs are fundamental to understanding how insect assemblages are structured because they limit the degree of similarity between guild members and thereby facilitate species coexistence [44]. This prediction could be tested through experimental manipulations of the various trade-offs associated with egg maturation strategy among members of a chosen parasitoid guild.

Our findings also have important implications for pest management. Parasitoid wasps occur in most terrestrial insect food webs [45]–[48] where they play a major role in the population dynamics of their hosts by constraining or driving population fluctuations and by limiting average population levels [49]–[50]. Consequently, they are widely used in biological control [47], [51]–[52], one method of which involves releasing a massive quantity of parasitoids such as trichogrammatids into a crop (‘inundative release’) to rapidly inflict high levels of host mortality, usually in the host’s egg stage [20]. Trichogrammatids, being small-bodied, are likely to be subject to a very high risk of premature death through desiccation (*e.g.*, see [22]), a hazard that is likely to become more prevalent in some agroecosystems, due to global warming. Significant genetic variation in the temporal pattern of egg maturation offers, via selective breeding, two valuable opportunities for biological control practitioners. By increasing the amount of positive skew in the age-specific oogenesis schedule, not only could the problem of premature wasp mortality due to high temperature be mitigated, but also the wasps’ maximum oviposition rate could be increased. *T. brassicae* is likely the most intensively reared wasp species used in inundative release programmes worldwide, and our findings provide the impetus for future research into artificial selection for ‘improved’ egg maturation characteristics in this and other commonly mass-produced species.

In summary, the genetic variation we have recorded provides a platform for undertaking artificial selection manipulations of the temporal pattern of egg maturation in parasitoid wasps. Such work would provide insights into the evolution and diversification not only of that particular trait but also of the other, correlated traits, and it would help to explain both why parasitoid wasps show such marked diversity in life-history [1], [53] and why the order Hymenoptera – the group to which most insect parasitoids belong – is one of the most speciose within the class Insecta [54].
